# Cost‐effectiveness analysis of pembrolizumab plus chemotherapy versus chemotherapy as the first‐line treatment for advanced esophageal cancer

**DOI:** 10.1002/cam4.5350

**Published:** 2022-10-21

**Authors:** Zhuo‐Miao Ye, Zhe Xu, Hao‐Lun Wang, Ying‐Yuan Wang, Ze‐Chang Chen, Qin Zhou, Xiang‐Ping Li, Ying‐Ying Zhang

**Affiliations:** ^1^ Department of Oncology Xiangya Hospital, Central South University Changsha Hunan China; ^2^ Department of Pharmacy, Xiangya Hospital Central South University Changsha Hunan China; ^3^ Graduate School of Guangxi Medical University Nanning China; ^4^ Xiangya Medical College Central South University Changsha Hunan China

**Keywords:** cost‐effectiveness, esophageal cancer, Markov model, pembrolizumab

## Abstract

**Objective:**

The KEYNOTE‐590 trial showed that individuals with advanced esophageal cancer who received Pembrolizumab in combination with chemotherapy as a first‐line regimen achieved a significant extension of survival. However, this treatment option increases the financial burden on patients and the economic benefits remain to be further evaluated.

**Methods:**

A Markov model was used to simulate 10‐year survival of patients with esophageal cancer from the perspective of United States (US) Medicare payers. We evaluated the economics of Pembrolizumab plus chemotherapy in the PD‐L1 positive score (CPS ≥10) and any PD‐L1 expression groups, respectively. We estimated total costs, quality‐adjusted life years (QALYs), and calculated incremental cost effectiveness ratios (ICERs). Sensitivity analyses were conducted to explore the impact of uncertainties on the results. Subgroup analysis was also performed.

**Results:**

The analysis results showed that the ICER for pembrolizumab plus chemotherapy versus chemotherapy alone was $293,513.17/QALYs in the any PD‐L1 expression group. This exceeded the threshold of willingness to pay ($150,000/QALYs). ICERs were most sensitive to the cost of pembrolizumab and the ICERs exceeded $150,000/QALYs in all subgroups.

**Conclusions:**

Evidence suggests that first‐line pembrolizumab in combination with chemotherapy is not a cost‐effective option for advanced esophageal cancer in the US, regardless of PD‐L1 expression status.

## Graphical abstract

Cost‐Effectiveness; Pembrolizumab; Oesophageal cancer; Markov model

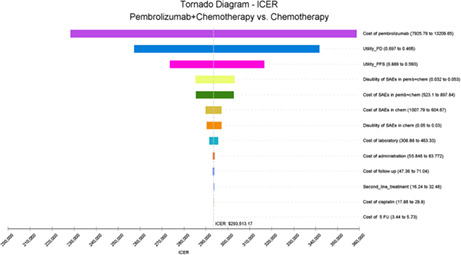

## 1 INTRODUCTION

Esophageal cancer is the eighth most common cancer globally and its incidence continue to increase annually. In 2019, approximately 17,000 people in the United States were diagnosed with esophageal cancer, accounting for nearly 1.0% of all new cases worldwide.^1^, ^2^ Esophageal adenocarcinoma is the most common histologic subtype in North America, and more men than women are affected.^2^ There are several risk factors for esophageal cancer, including age and smoking. The risk factors for esophageal adenocarcinoma are somewhat different from those for esophageal squamous cell carcinoma; in particular, gastroesophageal reflux (GERD), Barrett's esophagus (BE), obesity, and alcohol are more independent risk factors for esophageal adenocarcinoma.^3^ The current top‐line drug treatment intervention for patients with advanced, or recurrent esophageal cancer is fluorouracil plus platinum drugs; however, some patients still have poor survival improvement and present with further disease progression, and while drugs such as doxorubicin and paclitaxel have been attempted in such patients, the results remain unsatisfactory.^4^, ^5^, ^6^ This calls for a new and effective treatment regimen for patients whose disease course is still progressing after first‐line chemotherapy. Studies have confirmed that immune site inhibitors can enhance the anti‐tumor activity of immune cells by blocking the programmed cell death protein 1(PD‐1) pathway, and combining chemotherapy with immune checkpoint inhibitors (ICIs) is gradually becoming the preferred option for the treatment of various cancers.^7^, ^8^ In a phase 3 study (KEYNOTE‐590), the ICIplus chemotherapy for advanced esophageal cancer for the first‐line treatment showed better antitumor activity than chemotherapy alone. This combination treatment regimen significantly improved the prognosis and corresponding objective outcomes in patients with esophageal cancer.^9^

Although some literatures have demonstrated that pembrolizumab combined with chemotherapy has a greater therapeutic benefit than chemotherapy alone, it simultaneously carries a significant economic burden. Excessive treatment costs may prompt patients to abandon optimal treatment options; so, it is critical to strike a balance between cost and efficacy. This study, which is based on a phase III study (KEYNOTE‐590), evaluated the cost‐effectiveness of pembrolizumab plus chemotherapy versus fluorouracil plus platinum drugs as the first‐line treatment for advanced esophageal cancer.

## 2 MATERIALS AND METHODS

### 2.1 Population

The target cohort simulated by the model was assumed to be the population studied in the phase III KEYNOTE‐590 trial. PD‐L1 expression was determined in all patients by immunohistochemistry. Patients were then divided into three subgroups: a PD‐L1 combined positive score ≥ 10 (CPS≥10) group, a CPS < 10 group, and all randomized patients group (any PD‐L1 expression group). We performed a cost effectiveness analysis of pembrolizumab plus chemotherapy based on published survival data from the KEYNOTE‐590 trial for patients in the any PD‐L1 expression group and the CPS≥10 group.

### 2.2 Model structure

In this study, efficacy and safety data for the pembrolizumab plus chemotherapy regimen were collected from the KEYNOTE 590. A Markov model was built for the long‐term survival simulation of the target cohort. The model consists of three independent states: progression‐free survival (PFS), progressive disease (PD), and death. Details of the transfer between the various states of the model were shown in Figure S1. The model was run from the time individuals were randomly assigned to receive treatment. All individuals received pembrolizumab plus chemotherapy or chemotherapy in the PFS state, and as the model was run, individuals who failed first‐line treatment were transferred to the PD state.^10^ Nearly half of the patients received subsequent treatment (43.5% and 47.8% of patients in the intervention and control groups, respectively). We set the model cycle to 21 days to align with the individual's dosing cycle. The model was run for 10 years to simulate the entire life course of the target cohort (over 99% of individuals were observed to die).^11^ The Markov model was programmed and run using Treeage pro (version 2021). Referring to previous related studies, the discount rate for cumulative costs and health outputs was set at 3%.^12^ Primary study outcome was incremental cost‐effectiveness ratio (ICER), which was defined as the cost per additional quality‐adjusted life year (QALY). From the US healthcare payers' perspective, the willingness to pay (WTP) threshold was assumed to be $150,000/QALY.^13^ Comparing ICER to WTP thresholds to determine whether treatment options were cost‐effective. The study protocol was designed with reference to consolidated health economic evaluation reporting standards (CHEERS).^14^

### 2.3 Clinical data

The Kaplan–Meier (KM) survival curves published in the KEYNOTE‐590 trial were assumed to be the expected efficacy data for the intervention regimen of this study. The graphical digital extraction software Getdata (version 2.26) was used to obtain the survival data for the OS and PFS curves. Individual data at the virtual level were then generated using a program programmed with R software (version 4.2.1). The program reproduced the patient's clinical outcome data as closely as possible, as disclosed in a previous study by Guyot et al.^15^ The generated virtual individual data were mathematically fitted using parametric survival distributions including exponential, log‐logistic, log‐normal, Weibull and gamma.^16^ The flexsurv package in R software was used to estimate.

The goodness of fit of the parameter survival distribution (The log‐logistic distribution was considered to have the best fit to the survival data). Model fits and survival predictions were shown in Table S1 and Figure S2. The probability of transfer was estimated based on two specific parameters (shape and scale) of the log‐logistic distribution. The formula for the transfer probability was obtained from Liu et al.^17^

### 2.4 Utility estimates

Utility was used to measure quality of life across health states, ranging from 0 (death) to 1 (full health). During the follow‐up, the KEYNOTE‐590 trial used the European Five‐Dimensional Health Scale (EQ‐5D‐5L) to evaluate the patients' health quality. Because patient utility data from the KEYNOTE‐590 trial were not available, model inputs for utility were obtained from a previous study evaluating the economics of esophageal cancer.^18^ The utilities for PFS and PD states were 0.741 and 0.581, respectively.^19^ The safety of the treatment regimen mainly considers the loss of utility due to adverse events (AEs). The main three AEs resulting from the treatment were selected as model inputs for the treatment and intervention groups, respectively.^20^, ^21^

### 2.5 Cost estimates

Our medical costs inputs included the cost of pembrolizumab and chemotherapy (5‐fluorouracil and cisplatin), AEs disposal costs, disease management costs, follow‐up costs, post‐progression treatment costs, and laboratory testing costs. The drug prices were estimated to drug unit prices published by the Centers of Medicare Services in October 2021.^22^ Therefore, drug cost was the unit price of the drug multiplied by the actual clinical dose per treatment cycle. In the treatment plan of the KEYNOTE‐590 trial, patients received immunotherapy (pembrolizumab, 200 mg, intravenous injection) with or without chemotherapy (cisplatin, 80 mg/m^2^;5‐fluorouracil, 800 mg/m^2^) once every three weeks. We assumed that the average weight of a patient was 70 kg and that the average body surface area was 1.86 m^2^.^23^ Referring to a recently published article, we collected the treatment costs per AEs incurred.^20^, ^24^, ^25^, ^26^ To simplify the model, AEs were assumed to occur in the first cycle of the model. The NCCN treatment guidelines (version 2021) stated that after progression on first‐line treatment with immunotherapy or chemotherapy, patients were recommended to receive a combination chemotherapy regimen of oxaliplatin plus fluorouracil.^27^ We assumed that patients in the model were treated with this regimen for follow‐up after failure of first‐line therapy and estimated the cost per cycle of therapy. The baseline values of cost were inflated according to the 2022 US consumer price index.

### 2.6 Sensitivity analyses

To quantify the effect of model input parameters on the analysis results and to assess uncertainty, we performed sensitivity analysis. The fluctuation ranges of the parameters analyzed were determined from previous literature. It is assumed that these parameters change within ±25% of the baseline value.^28^ The baseline values, upper and lower limits and distributions of the model input parameters were shown in Table 1. We also performed a probabilistic sensitivity analysis (PSA) to explore the probability that pembrolizumab plus chemotherapy was cost‐effective across all model input parameter variations. In the Monte Carlo simulation with 10,000 random samples, health utility, and AE incidence were defined as beta distributions, and treatment‐related costs were defined as gamma distributions. The cost‐effectiveness acceptability curve (CEAC) was used to explore the relationship between the likelihood of pembrolizumab plus chemotherapy being cost‐effective and the WTP threshold.

**TABLE 1:** Model parameters: baseline values, ranges, and distributions for sensitivity analysis

	Baseline value	Lower limit	Upper limit	Distribution	Reference
Log‐logistic survival model of pembrolizumab + chemotherapy in APE group					
PFS	γ = 1.815 μ = 6.699	–	–	–	Model fitting
OS	γ = 1.676 μ = 13.033	–	–	–	Model fitting
					Model fitting
Log‐logistic survival model of chemotherapy in APE group					
PFS	γ = 2.089	–	–	–	Model fitting
	μ = 4.825	–	–	–	Model fitting
OS	γ = 1.822	–	–	–	Model fitting
	μ = 9.810	–	–	–	Model fitting
Drug cost, $/per cycle					
Pembrolizumab	10567.72	7925.79	13209.65	Gamma	CMS^22^
5‐FU	4.58	3.44	5.73	Gamma	CMS^22^
Cisplatin	23.84	17.88	29.80	Gamma	CMS^22^
Second‐line treatment	21.65	16.24	32.48	Gamma	NCCN guidelines^27^
Follow‐up cost per cycle	59.2	44.40	74.00	Gamma	^25^
Laboratory per cycle	386.12	308.88	463.33	Gamma	^26^
Administration per cycle	69.81	55.848	83.772	Gamma	^25^
Expenditures on main SAEs, $/ per cycle					
Pembrolizumab + chemotherapy	697.47	523.10	897.84	Gamma	19, 24
Chemotherapy	806.23	604.67	1007.79	Gamma	19, 24
Health utilities					
PFS	0.741	0.593	0.889	Beta	^18^
PD	0.581	0.465	0.697	Beta	^18^
Disutility due to SAEs					
Pembrolizumab + chemotherapy	0.042	0.032	0.053	Beta	20, 21
Chemotherapy	0.040	0.030	0.050	Beta	
Death	0	–	–	–	

Abbreviations: APE, Any PD‐L1 expression group; PD, Progressive disease; PFS, Progression‐free survival; SAE, Severe adverse event.

Based on the subgroup forest chart published in the KEYNOTE‐590 clinical trial, subgroup analysis was conducted in any PD‐L1 expression group. We assumed that the baseline characteristics of each chemotherapy subgroup were the same as that in the PFS and OS curve of the total chemotherapy population, and the OS and PFS rate of pembrolizumab plus chemotherapy was estimated by subgroup‐specific hazard ratios (HRs).^10^, ^29^

In order to investigate the relationship between the choice of model extrapolation method to the results of the analysis, we used exponential, Weibull, and log‐normal parametric distributions to fit the KM survival curves of the any PD‐L1 expression group.

## 3 RESULTS

### 3.1 Base‐case analysis

Table 2 indicated that the incremental cost of pembrolizumab plus chemotherapy in the any PD‐L1 expression group was $164,876.52. The incremental health outputs were 1.04 LYs and 0.56 QALYs. The ICER for pembrolizumab plus chemotherapy was $293,513.17 per QALY in the any PD‐L1 expression group. Additionally, the ICER per QALY in the CPS≥10 group was $294,000.03 per QALY. The detailed analysis of the results is presented in Table S4.

**TABLE 2:** Base‐case analysis results

Strategies	Cost	Incr Cost	LYs	Incr LYs	ICER/ LYs	QALYs	Incr QALYs	ICER/ QALYs
Chemotherapy	13991.31		0.66			0.45		
Pembrolizumab + Chemotherapy	178867.73	164876.42	1.71	1.04	159877.76	1.01	0.56	293513.17

Abbreviations: ICER, Incremental cost‐effectiveness ratio; Incr Cost, Incremental cost; Incr Lys, Incremental life‐years; Incr QALYs, Incremental *Quality*‐adjusted life‐years; Lys, life‐years; QALYs, Quality‐adjusted life‐years.

### 3.2 Sensitivity analyses

**FIGURE 1:**
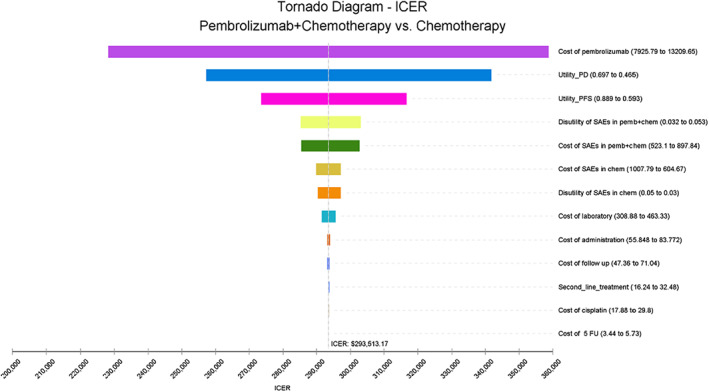
Tornado diagram in any PD‐L1 expression group. ICER, incremental cost‐effectiveness ratio; PD, progressive disease; PFS, progression‐free survival; QALY, quality‐adjusted life years.

**FIGURE 2:**
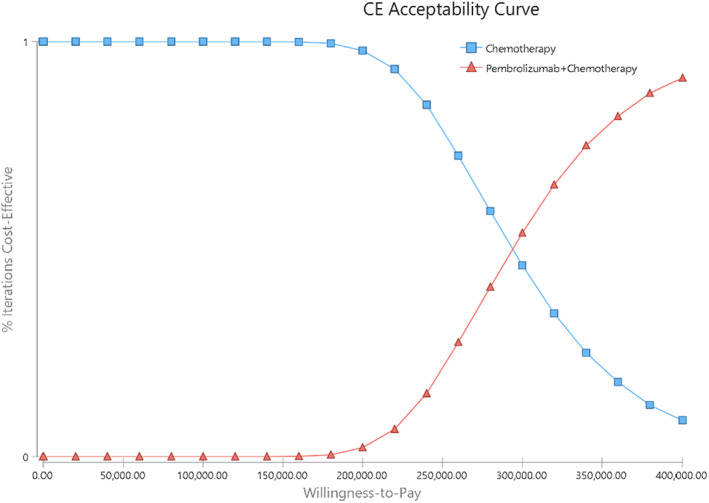
Acceptability Curves for the choice of pembrolizumab plus chemotherapy versus chemotherapy at different WTP thresholds in any PD‐L1 expression group. WTP, willingness‐to‐pay.

The tornado diagram (Figure 1) showed the ranking of the effects of uncertainty in the model input parameters. The input parameters of each model were arranged in descending order according to their influence on ICER, and the degree of influence was represented by the width of the bar chart. The analysis results demonstrated that in the any PD‐L1 expression group, the cost of pembrolizumab, utility of PD, and PFS had the greatest influence on the ICER. The price of pembrolizumab was among the most influential factors; therefore, we performed a scenario analysis about the price of pembrolizumab (Figure S4). When pembrolizumab cost was less than $4762.09 per cycle (or at 54.9% less the cost), the ICER would be below $150,000 /per QALY. The results of the PSA were explained by the incremental cost–benefit scatter chart (Figure S5) and CEAC (Figure 2). Figure S5 indicated that all the simulation points were above the WTP line, and Figure 2 demonstrated that the probability of pembrolizumab plus chemotherapy being cost‐effective was 0.4% in all randomized patient groups. To find potential cost‐effective patient subgroups, we performed subgroup analyses of the total population according to the survival rate of patients in KEYNOTE‐590 (Table S5). Unfortunately, none of these subgroups was cost‐effective.

The results of fitting and extrapolating the OS and PFS curve data based on different parameter distributions are presented in Table S6. The model results were influenced to some extent by the choice of the extrapolation method for the survival curves. Fitting with a Log‐normal distribution yielded the smallest ICER of $286,855.65 per QALY. However, the result was still not cost‐effective.

## 4 DISCUSSION

KEYNOTE‐590 illustrates the significant clinical benefit of pembrolizumab combined with chemotherapy, which is a breakthrough in the treatment of esophageal cancer.^9^ However, the financial expenses associated with long‐term immunotherapy exceeds the medical financial burden that families with ordinary income can bear. How to find a balance between cost and benefit is still an important issue worth exploring.

In October 2016, the FDA approved pembrolizumab monotherapy for metastatic NSCLC.^30^ However, many patients cannot bear the financial burden imposed by immunotherapy (approximately $13,000 per month).^31^ This may discourage clinicians from administering immunotherapy, limiting its use to affluent populations. The income level of patients is often used to determine the kind of treatment administered.^32^ Due to the specific nature of market licensing and medical reimbursement, the cost of anticancer drugs may be an urgent issue in the United States.^33^ Therefore, an evaluation of immunotherapy, such as pembrolizumab, could help in avoiding the wastage of healthcare resources. It could also guide physicians in selecting the best treatment options for patients.

Previous literatures have researched the economics of pembrolizumab in lung cancer patients.^30^, ^34^ However, there still lacks an appropriate assessment of pembrolizumab for advanced esophageal cancer as the first‐line treatment. Therefore, the assessment of the cost and health outcomes of pembrolizumab is necessary to assess whether this drug has the potential for widespread dissemination and usage.

We established the model to evaluate the economics of pembrolizumab combined with chemotherapy verus chemotherapy according to KEYNOTE‐590 in the perspective of US healthcare payers. Unfortunately, we only obtained the data of the CPS≥10 group and all randomized patients through the KEYNOTE‐590 trial. In our analysis, pembrolizumab plus chemotherapy was not cost‐effective in both the CPS ≥10 group and in all randomized patient groups. It is worth mentioning that the lack of economic benefits cannot be the only factor affecting the clinical use of pembrolizumab.^35^ Clinicians should seek a balance between drug toxicity, efficacy, and economic benefits, despite the high cost of the new immunological drugs.

Reducing the cost of ICI, particularly pembrolizumab, can effectively reduce the burden of disease on patients receiving treatment, which is an important discussion point. We found that if the cost of pembrolizumab was reduced by 54.9% or more, the ICER was below $150,000/QALY. The efficacy of pembrolizumab plus chemotherapy varies between different patient subgroups; however, these differences cannot reverse the results of the cost‐effectiveness analysis. Changing the price of pembrolizumab was still the most effective and feasible strategy to increase its economic benefits. The findings from our study are useful for local healthcare decision‐makers and in resource allocation for esophageal cancer treatment.

Several studies have analyzed the cost‐effectiveness about pembrolizumab in esophageal cancer. Qu et al. investigated the pembrolizumab plus chemotherapy in patients with advanced esophageal cancer using a partitioned survival model and showed that the ICER was $118,875/QALY.^36^ They came to the conclusion that pembrolizumab would be cost‐effective. Their research was different in several respects: (1) they used a partitioned survival model, which may make some differences; (2) they made a 20% cost‐sharing assumption; and (3) they modeled utilities based on EuroQol‐five dimension scale (EQ‐5D) data; however, this part of the data is difficult to obtain. In addition, according to their study, the choice of different extrapolation methods for overall survival may create significant uncertainty in the results of the analysis. In our study, we analyzed several distributions commonly used in economic decision analysis; however, this only had limited impact on the ICER. Other PD‐L1 inhibitors were also less cost‐effective than chemotherapy when treating the esophageal cancer. In the analysis by Zhang et al, model analysis based on nivolumab regimen results far exceeded the WTP threshold in China ($136,709/QALY vs $29,306/QALY).^19^ Therefore, for economic reasons, nivolumab is not recommended as a second‐line treatment option. Overview, at current prices in the United States, PD‐L1 inhibitors are not a recommended treatment for patients with esophageal cancer.

## 5 LIMITATIONS

Our study has limitations. First, we used a model to simplify the KEYNOTE‐590 clinical trial because of a lack of sufficient data. Only major AEs were selected. Second, due to the limited sample size of KEYNOTE‐590 and the insufficient follow‐up time, and we could not obtain the real follow‐up data, we could only predict the survival of patients in the later stage through the extrapolation of the model. Third, our PFS and PD utility were obtained from previously published relevant literatures. Fourth, to simplify the model, we only considered the major adverse effects and their cost impacts and utility changes, and the specific data were referred to previous published articles. Fifth, unfortunately, we did not find specific subsequent therapy options according to KEYNOTE‐590. We referred to the guidelines and assumed that patients in the different groups would receive the same subsequent therapy after disease progression, which would have affected the treatment effect in the two groups.

## 6 CONCLUSION

From the US healthcare payers' perspective, compared with chemotherapy and regardless of the PD‐L1 expression status, pembrolizumab plus chemotherapy as the first‐line treatment in patients with locally advanced/unresectable or metastatic esophageal cancer is not cost‐effective at a WTP threshold of $150,000/QALY.

## AUTHOR CONTRIBUTIONS

**Zhuo‐miao Ye:** Conceptualization (lead); data curation (lead); formal analysis (lead); investigation (lead); methodology (lead); project administration (lead); writing – original draft (lead); writing – review and editing (lead). **Zhe Xu:** Conceptualization (lead); data curation (lead); formal analysis (lead); investigation (lead); methodology (lead); project administration (lead); writing – original draft (lead); writing – review and editing (lead). **Hao‐lun Wang:** Data curation (supporting); formal analysis (supporting); methodology (supporting); project administration (supporting); writing – original draft (supporting). **Ying‐yuan Wang:** Data curation (supporting); investigation (supporting); methodology (supporting); project administration (supporting). **Ze‐chang Chen:** Conceptualization (supporting); formal analysis (supporting); investigation (supporting); methodology (supporting); project administration (supporting). **Qin Zhou:** Investigation (supporting); methodology (supporting); project administration (supporting); software (lead); supervision (supporting). **Xiang‐Ping Li:** Conceptualization (equal); funding acquisition (lead); supervision (lead); validation (lead); visualization (lead). **Ying‐ying Zhang:** Conceptualization (equal); funding acquisition (lead); validation (lead); visualization (lead).

## CONFLICT OF INTEREST

The authors declare that they have no competing interests.

## ETHICS APPROVAL AND CONSENT TO PARTICIPATE

Not applicable.

## PATIENT CONSENT FOR PUBLICATION

Not applicable.

## Supporting information


Figure S1‐S5
Table S1‐S6

## Data Availability

The datasets generated and analyzed during the present study are available from the corresponding author on reasonable request.
