# Supplementary material for: Cost‐effectiveness analysis of pembrolizumab plus chemotherapy versus chemotherapy as the first‐line treatment for advanced esophageal cancer
Source: Cancer Med. 2022 Oct 21;12(5):6182–9. doi: 10.1002/cam4.5350 (PMC10028044; doi:10.1002/cam4.5350)
Supplement: Supplementary file 1 — Figure S1‐S5 Table S1‐S6 [file CAM4-12-6182-s001.docx]

eFigure1: Markov state transition probability diagram.


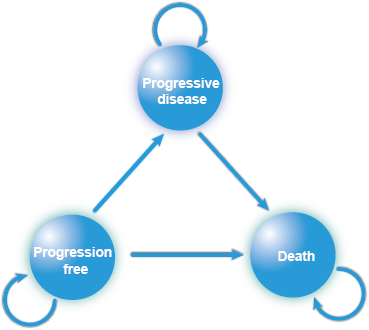


eFigure 2. Fitting and extrapolation of the Kaplan Meier survival curve


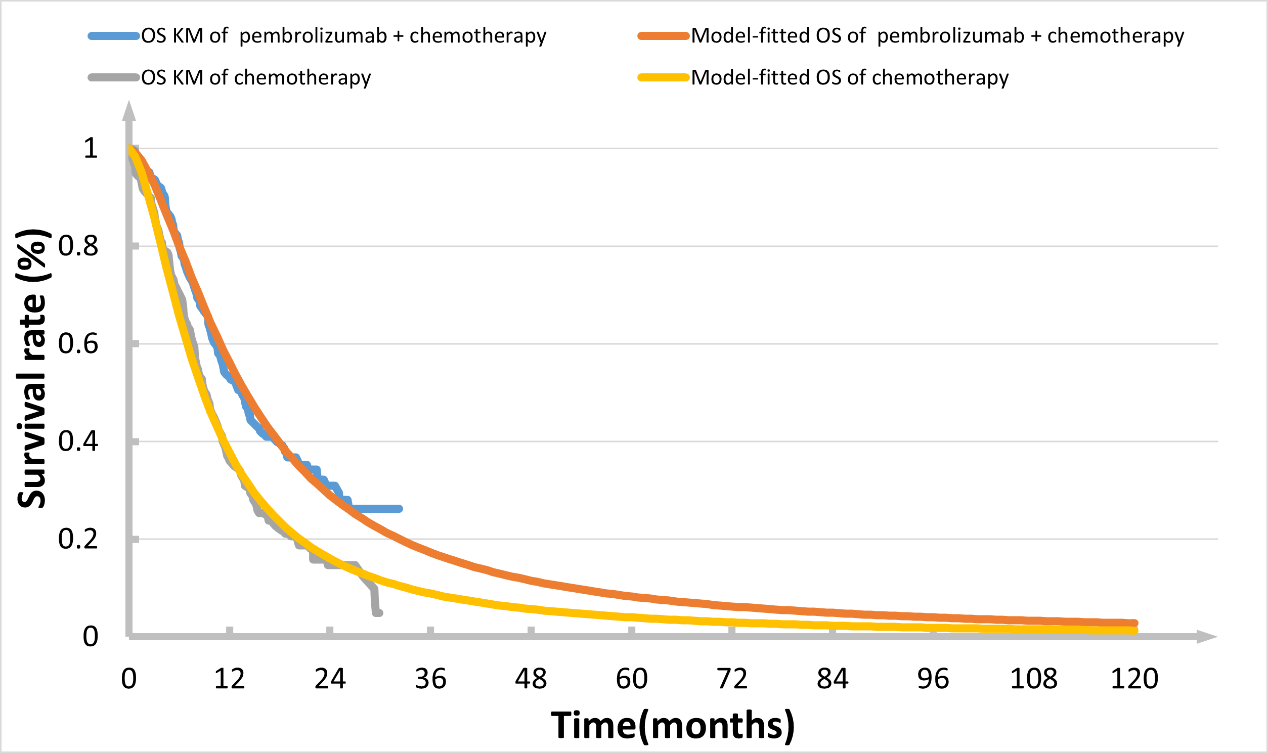


1. Model-fitted versus original OS curve in CPS≥10 group.


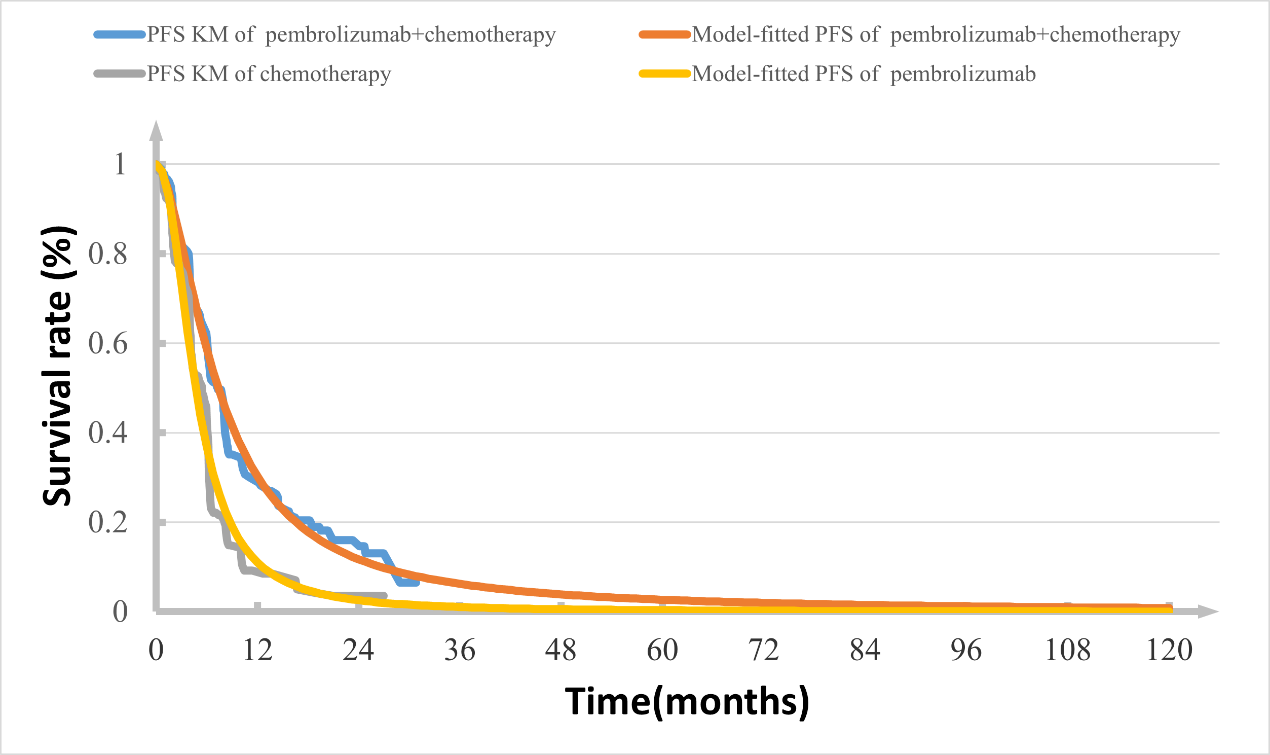


1. Model-fitted versus original PFS curve in the CPS≥10 group.


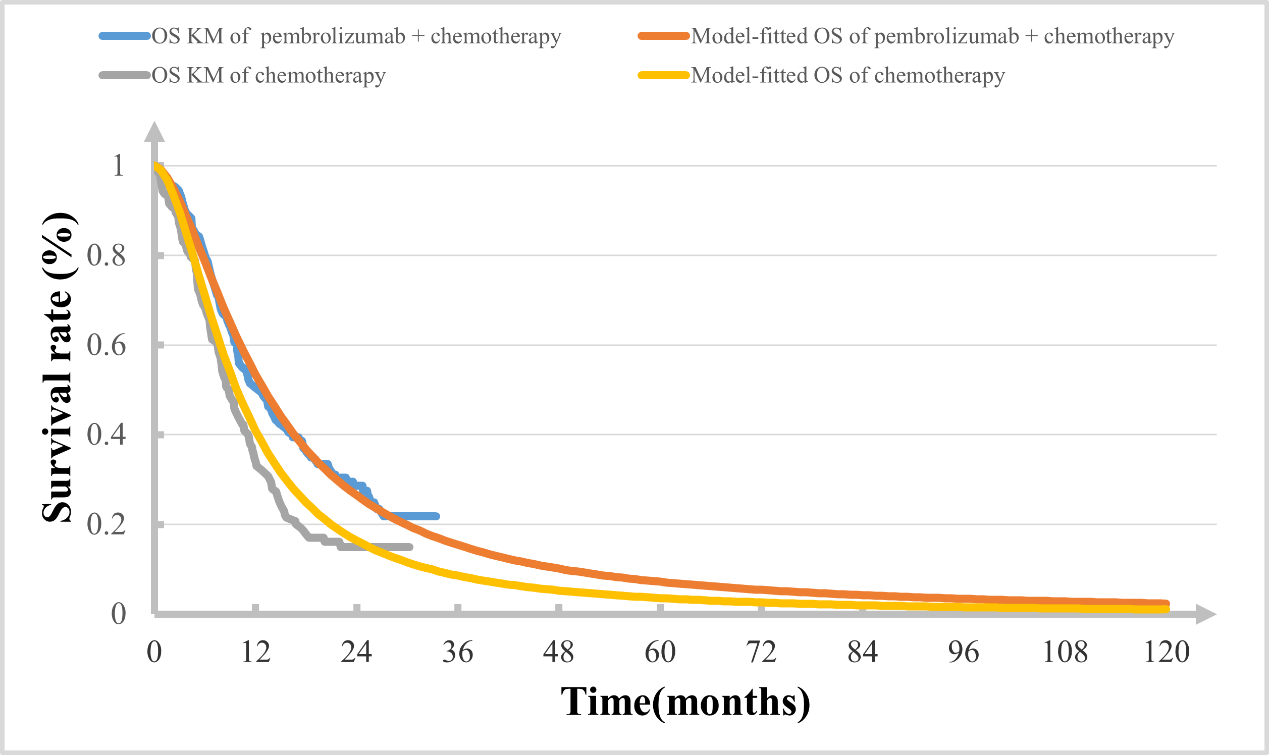


C. Model-fitted versus original OS curve in any PD-L1 expression group.


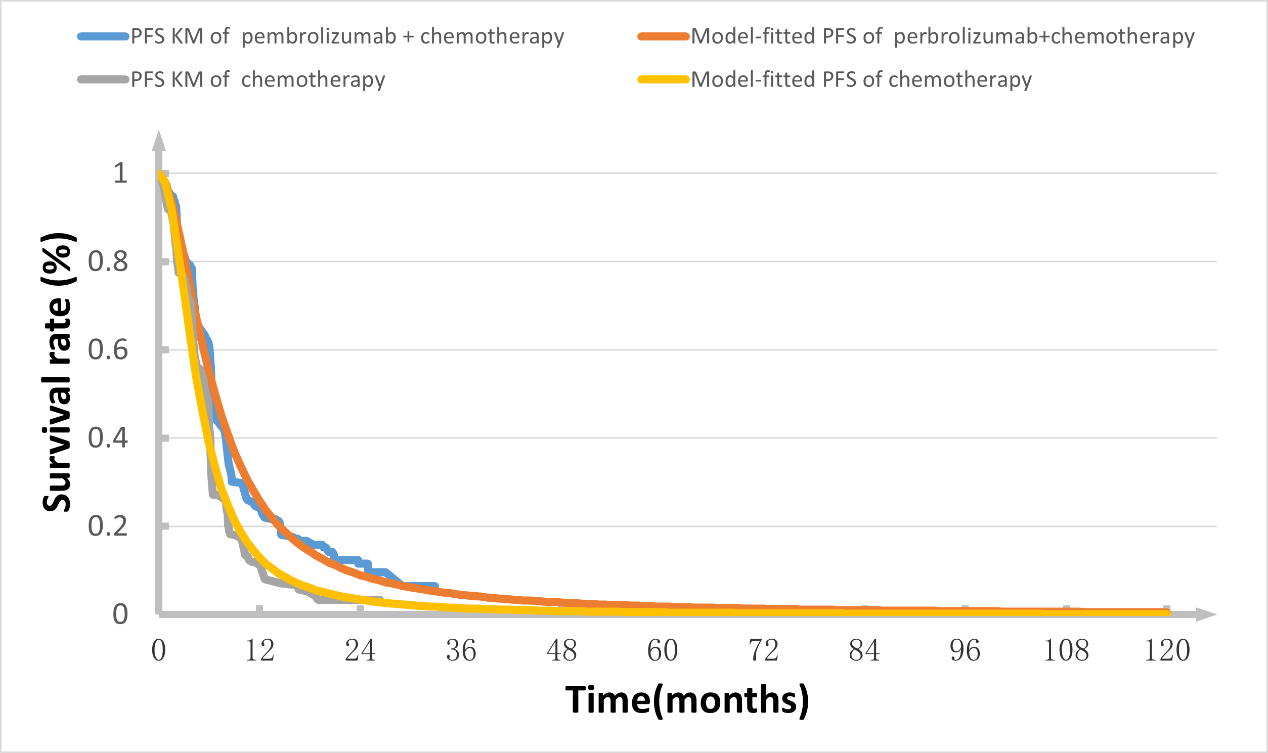


D. Model-fitted versus original PFS curve in any PD-L1 expression group.

efigure 2 is the extrapolation of the model results using log logistic distribution. It can be seen that the long-term fitting of OS and PFS data is still very reasonable.

Abbreviation: PFS= Progression-free survival; PD= Progressive disease; OS= Overall Survival; KM= Kaplan Meier survival curve.

eFigure3. Tornado diagram for one-way sensitivity analysis in the CPS≥10 group


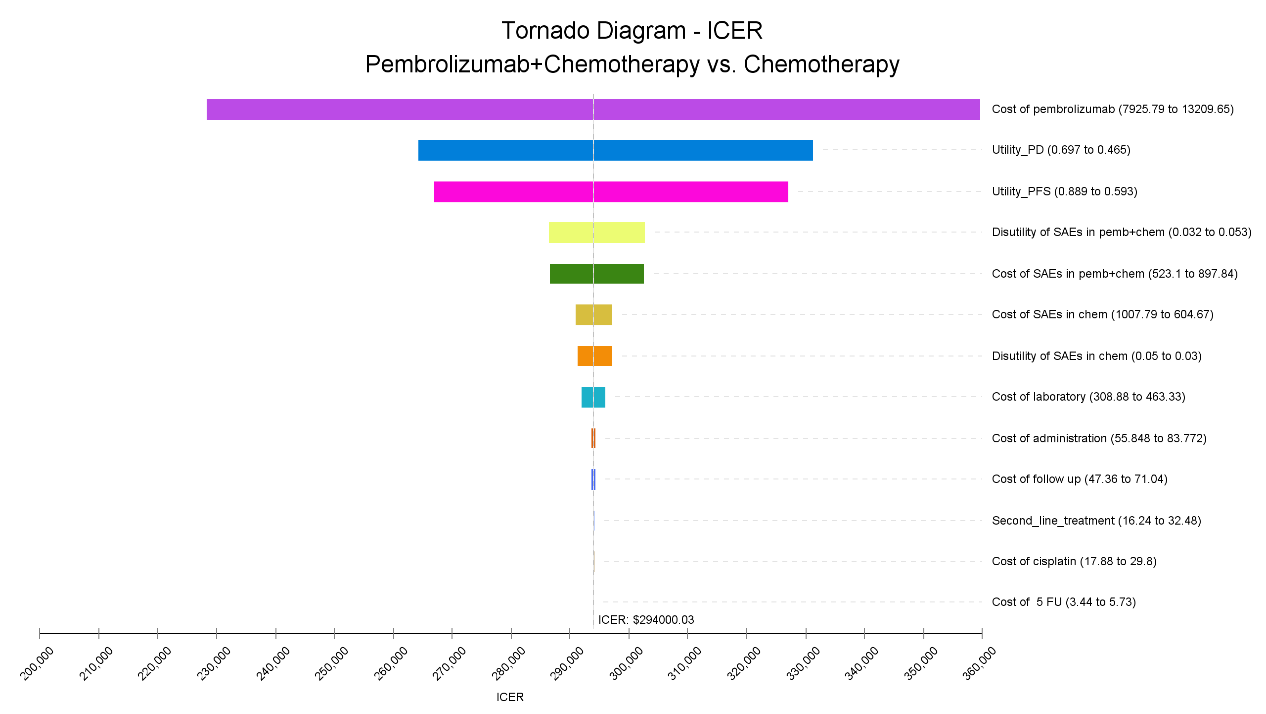


The results of tornado diagram showed that in the CPS ≥ 10 group, the cost of pembrolizumab, the utility of PD and the utility of PFS were the most influential factors on the analysis results, which was consistent with the results of any PD-L1 expression group.

Abbreviations: chem = chemotherapy; pemb+chem = pembrolizumab + chemotherapy; SAEs= serious adverse events; PD=progressive disease; PFS=progression-free survival; ICER=incremental cost-effectiveness ratio; QALY=quality-adjusted life years.

eFigure4. One-Way sensitivity analysis of the cost of pembrolizumab

(A)


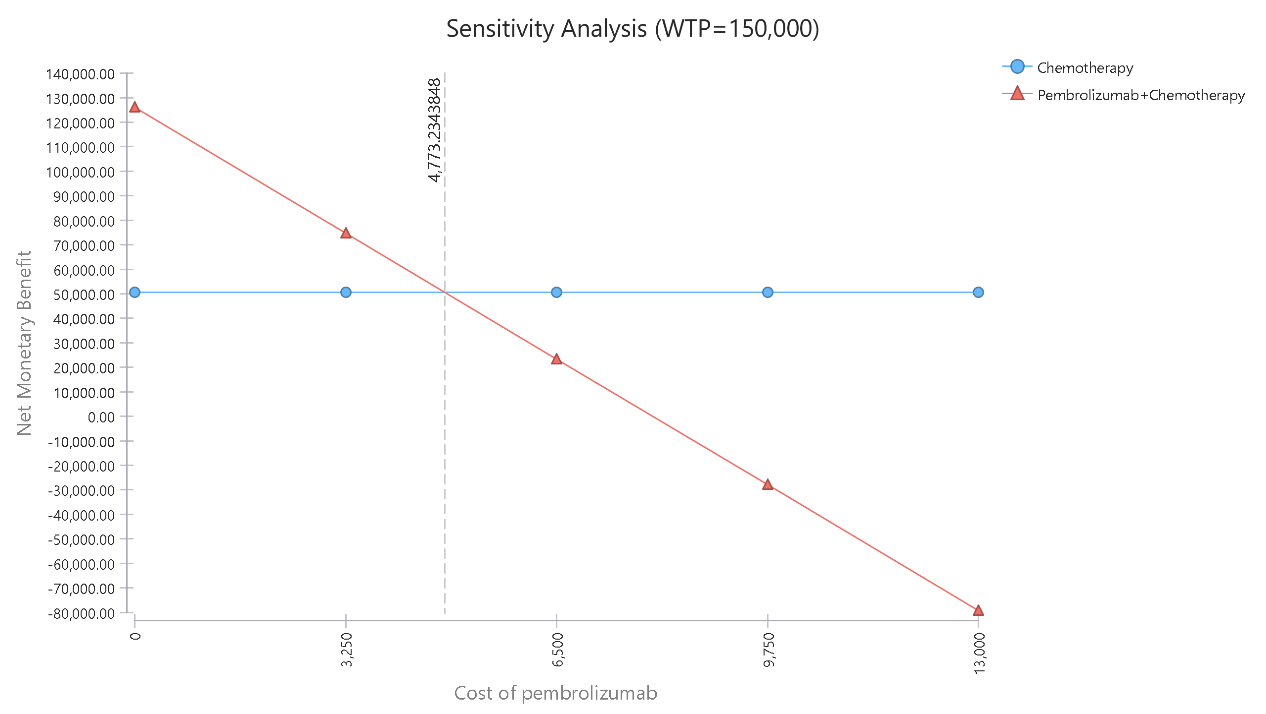


(B)


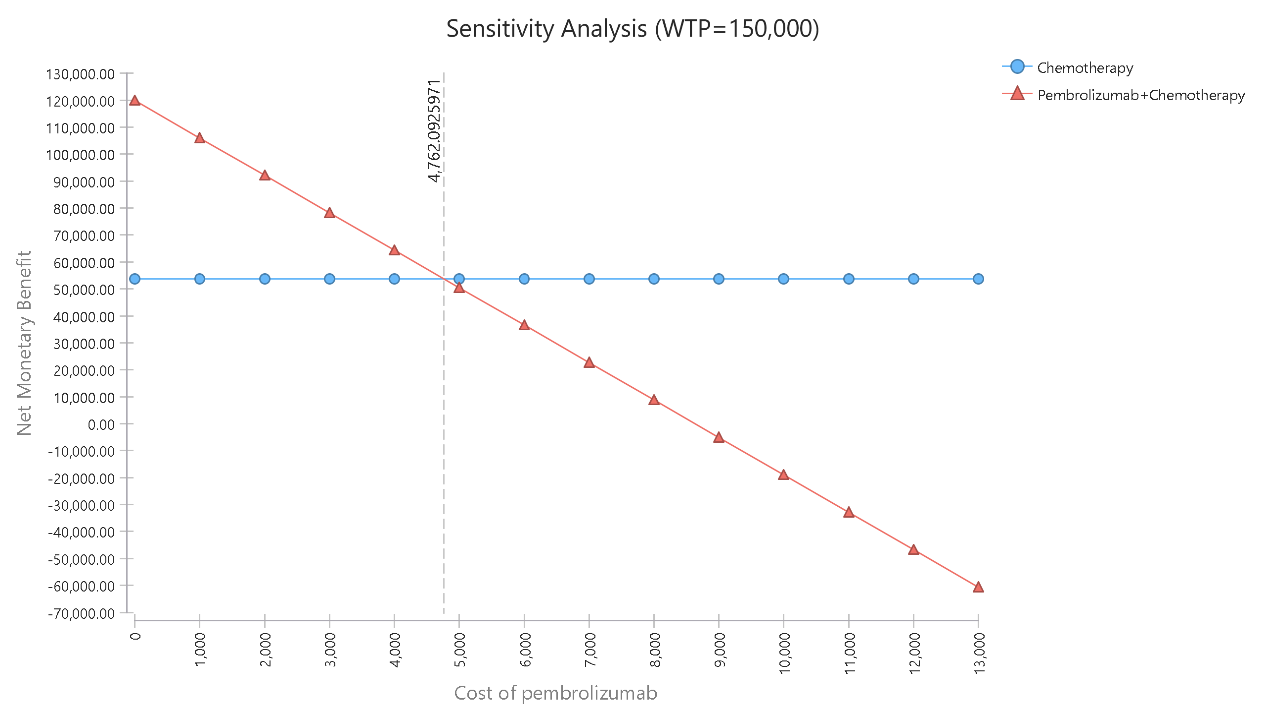


eFigure4. One-Way sensitivity analysis of the cost of pembrolizumab. (A) in CPS≥10 group; (B) in any PD-L1 expression group.

eFigure5.Incremental Cost-effectiveness Scatter Plot (Pembrolizumab plus chemotherapy vs Chemotherapy).

(A)


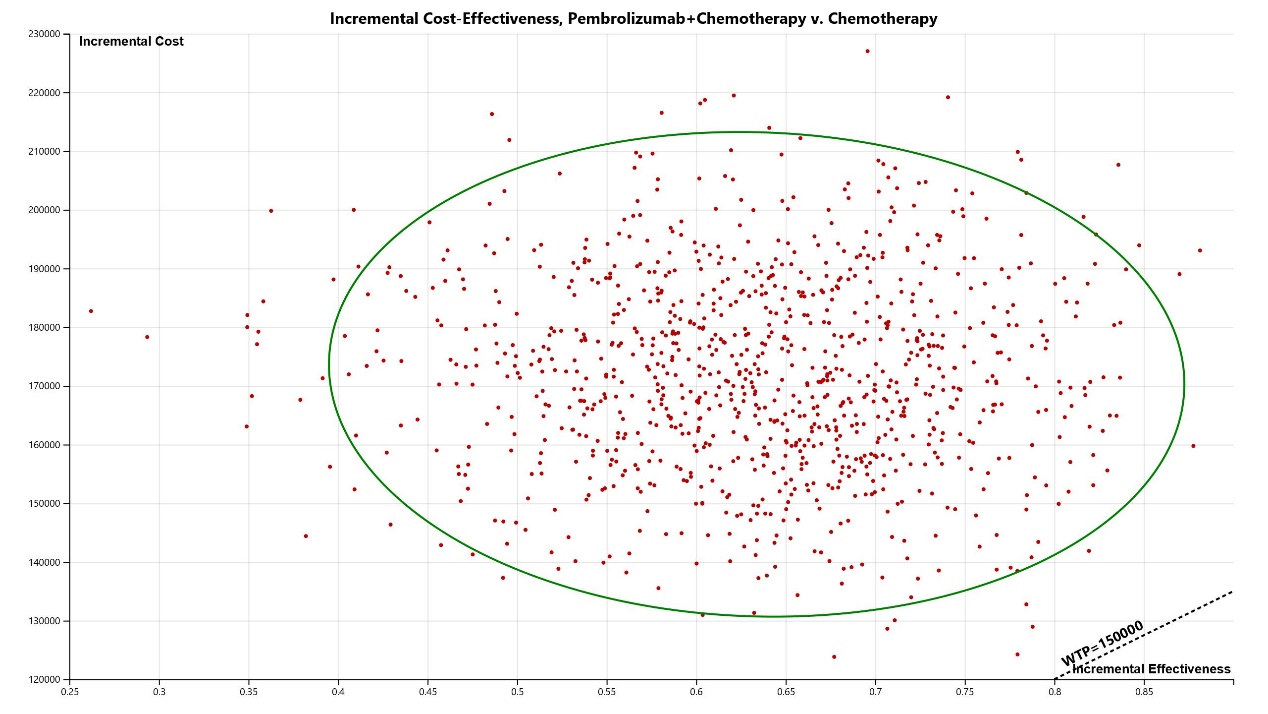


(B)


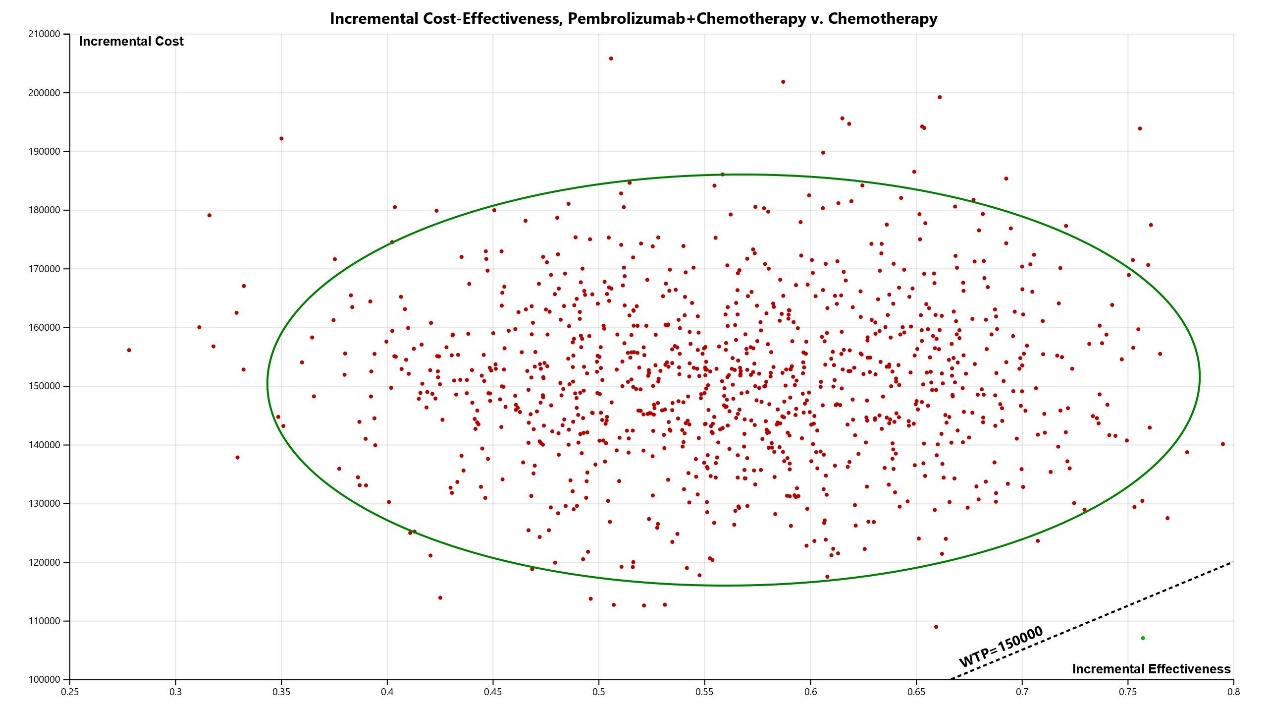


eFigure5.Incremental Cost-effectiveness Scatter Plot (Pembrolizumab + chemotherapy vs Chemotherapy). (A) in CPS≥10 group; (B) in any PD-L1 expression group.

ICER: Incremental cost-effectiveness ratio; WTP: Willingness-to-pay; QALY: Quality-adjusted life year.

eTable1.AIC and BIC scores of the fitted distribution in the CPS≥10 group and in the any PD-L1 expression group

| Distribution | OS of pembrolizumab + chemotherapy | | | OS of chemotherapy | | PFS of pembrolizumab + chemotherapy | | PFS of chemotherapy | |
| --- | --- | --- | --- | --- | --- | --- | --- | --- | --- |
| **CPS≥10 group** | AIC | BIC | AIC | | BIC | AIC | BIC | AIC | BIC |
| Exponential | 1012.854 | 1019.305 | 1201.311 | | 1207.877 | 991.2135 | 997.665 | 1029.874 | 1036.44 |
| Gamma | 1004.69 | 1014.367 | 1195.127 | | 1204.976 | 985.2276 | 994.9049 | 998.2358 | 1008.085 |
| Weibull | 1007.329 | 1017.007 | 1195.609 | | 1205.459 | 989.012 | 998.6892 | 1008.386 | 1018.235 |
| Log-normal | 999.4034 | 1009.081 | 1213.868 | | 1223.717 | 970.937 | 980.6142 | 994.2872 | 1004.137 |
| Log-logistic | **998.1324** | **1007.81** | **1199.04** | | **1208.89** | **970.1678** | **979.845** | **983.3494** | **993.199** |
| **Any PD-L1 expression group** | AIC | BIC | AIC | | BIC | AIC | BIC | AIC | BIC |
| Exponential | 1508.686 | 1515.913 | 1631.503 | | 1638.729 | 1468.623 | 1475.849 | 1460.726 | 1467.952 |
| Gamma | 1494.952 | 1505.791 | 1611.737 | | 1622.576 | 1455.984 | 1466.824 | 1424.003 | 1434.842 |
| Weibull | 1498.361 | 1509.187 | 1616.243 | | 1627.082 | 1462.51 | 1473.349 | 1431.855 | 1442.694 |
| Log-normal | 1494.75 | 1505.59 | 1615.875 | | 1626.714 | 1444.648 | 1455.487 | 1436.54 | 1447.379 |
| Log-logistic | **1486.559** | **1497.398** | **1602.665** | | **1613.505** | **1430.836** | **1441.675** | **1418.937** | **1429.777** |

The results showed that the Log-logistic distribution had the lowest AIC and BIC values, so we used Log-logistic distribution to fit the OS and PFS curves in our analysis.

Abbreviation: CPS≥10: PD-L1 combined positive score of 10 or more; PD-L1: Programmed cell death-Ligand 1; OS=Overall survival; PFS= Progression-free survival; AIC= Akaike information criterion; BIC= Bayesian information criterion.

eTable 2. Associated Costs of Grade 3 to 4 Treatment-Related Adverse Events and second-line therapy.

|  | Incidence rate (%)^a^ | Baseline value^b^ | Reference |
| --- | --- | --- | --- |
| **Cost of** **SAEs, per cycle** |  |  |  |
| **Pembrolizumab + chemotherapy** |  |  |  |
| Anaemia | 12 | 4285.92 | ^[24]^ |
| Decreased neutrophil count | 23 | 494.99 | ^[19]^ |
| Neutropenia | 14 | 494.99 | ^c^ |
| **Chemotherapy** |  |  |  |
| Anaemia | 15 | 4285.92 | ^[24]^ |
| Decreased neutrophil count | 17 | 494.99 | ^[19]^ |
| Neutropenia | 16 | 494.99 | ^c^ |
| Cost of second-line treatment, per cycle |  |  |  |
| Oxaliplatin | - | 11.35 | ^d^ |
| 5-FU | - | 10.30 | ^d^ |

^a^Refers to the statistical data on the incidence of adverse events published by keynote-590 trial.

^b^Additional costs incurred when adverse events occur in each cycle (3 weeks).

^c^ Assumed to be the same as decreased neutrophil count.

^d^ The second-line treatment uses a combination therapy regimen of oxaliplatin + 5-fluorouracil, with a two-week dosing cycle. Medication cost adjusted to 3 weeks according to model.

Abbreviation: SAEs= serious adverse events; 5-FU=5-fluorouracil.

eTable 3. Disutility from Grade 3 to 4 Treatment-Related Adverse Events

|  | Incidence rate (%) | Baseline value | Reference |
| --- | --- | --- | --- |
| **Pembrolizumab + chemotherapy** |  |  |  |
| Anaemia | 12 | 0.073 | ^[20]^ |
| Decreased neutrophil count | 23 | 0.090 | ^[21]^ |
| Neutropenia | 14 | 0.090 | ^a^ |
| **Chemotherapy** |  |  |  |
| Anaemia | 15 | 0.073 | ^[20]^ |
| Decreased neutrophil count | 17 | 0.090 | ^[21]^ |
| Neutropenia | 16 | 0.090 | ^a^ |

^a^Assumed to be the same as decreased neutrophil count.

eTable 4 Base-case analysis result in the CPS≥10 group.

| **Strategies** | **Cost** | **Incr Cost** | **LYs** | **Incr LYs** | **ICER/ LYs** | **QALYs** | **Incr QALYs** | **ICER/ QALYs** |
| --- | --- | --- | --- | --- | --- | --- | --- | --- |
| **CPS≥10 group** |  |  |  |  |  |  |  |  |
| Chemotherapy | 13277.33 |  | 0.63 |  |  | 0.43 |  |  |
| Pembrolizumab + Chemotherapy | 200097.08 | 186819.75 | 1.77 | 1.14 | 163708.06 | 1.06 | 0.64 | 294000.03 |

In the CPS ≥ 10 group, the ICER / QALYs of pembrolizumab + chemotherapy was $294,000.03 compared with chemotherapy, which was very close to the results obtained by any PD-L1 expression group.

Abbreviation: Incr Cost=Incremental cost; Lys=life-years; Incr Lys=Incremental life-years; QALYs= Quality-adjusted life-years; Incr QALYs=Incremental Quality-adjusted life-years; ICER= Incremental cost-effectiveness ratio; CPS≥10=PD-L1 combined positive score of 10 or more; PD-L1=Programmed cell death-Ligand 1.

eTable 5. Results of subgroup analyses in all randomized patients.

| **Subgroup** | **OS HR (95% CI)** | **PFS HR (95% CI)** | **ICER per QALY (95% CI)** | **Cost-effectiveness probability at WTP $150 000/QALY** |
| --- | --- | --- | --- | --- |
| Age |  |  |  |  |
| <65years | 0·76 (0·61–0·95) | 0.69 (0.56 - 1.08) | 225124.06(185606.53 to 268001.55) | 1.4% |
| ≥65years | 0·69 (0·53–0·89) | 0.62(0.48-0.80) | 197150.14 (169696.46 to 256848.13) | 2.2% |
| Sex |  |  |  |  |
| Female | 0·89 (0·59–1·35) | 0·74 (0·49–1·12) | 261330.44(176550.68 to 678748.53) | 0% |
| Male | 0·70 (0·58–0·84) | 0·63 (0·53–0·75) | 212471.22(193789.92 to 247115.24) | 2.1% |
| ECOG |  |  |  |  |
| 0 | 0·72 (0·55–0·94) | 0·57 (0·45–0·74) | 220750.23 (182419.07 to 275613.63) | 0.4% |
| 1 | 0·73 (0·59–0·90) | 0·71 (0·58–0·87) | 215556.75 (178810.08 to 258672.17) | 1.3% |
| Geographic region |  |  |  |  |
| Asia | 0·64 (0·51–0·81) | 0·59 (0·47–0·73) | 201156.53 (186974.64 to 243905.35) | 4.6% |
| Non-Asia | 0·83 (0·66–1·05) | 0·70 (0·56–0·89) | 243634.19 (209278.50 to 334340.67) | 0.1% |
| Histologic features |  |  |  |  |
| Adenocarcinoma | 0·74 (0·54–1·02) | 0·63 (0·46–0·87) | 222143.72(194580.43 to 318467.86) | 0.9% |
| Squamous cell carcinoma | 0·72 (0·60–0·88) | 0·65 (0·54–0·78) | 216332.89 (197027.57 to 260361.05) | 1.8% |
| PD-L1 status |  |  |  |  |
| CPS>10 | 0·62 (0·49–0·78) | 0·51 (0·41–0·65) | 204583.85 (192690.49 to 231209.84) | 3.8% |
| CPS <10 | 0·86 (0·68–1·10) | 0·80 (0·64–1·01) | 255030.57 (206959.99 to 392912.90) | 0% |

Abbreviation: CI, confidence interval; ICER, incremental cost-effectiveness ratio; OS HR, overall survival hazard ratio; PD-L1, programmed death ligand 1; QALY, quality-adjusted life-year; CPS, Combined Positive Score; WTP, Willingness-to-pay.

eTable 6 Base-case analysis results of fitting models with different parameter distributions in the any PD-L1 expression group

| **Distributions** | **Cost** | **Incr Cost** | **LYs** | **Incr LYs** | **ICER/ LYs** | **QALYs** | **Incr QALYs** | **ICER/ QALYs** |
| --- | --- | --- | --- | --- | --- | --- | --- | --- |
| **Exponential** |  |  |  |  |  |  |  |  |
| Chemotherapy | 16370.28 |  | 0.62 |  |  | 0.42 |  |  |
| Pembrolizumab + Chemotherapy | 174519.40 | 158149.12 | 1.60 | 0.98 | 162178.50 | 0.95 | 0.53 | 297158.19 |
| **Weibull** |  |  |  |  |  |  |  |  |
| Chemotherapy | 15956.07 |  | 0.60 |  |  | 0.41 |  |  |
| Pembrolizumab + Chemotherapy | 167207.60 | 151251.52 | 1.45 | 0.85 | 178655.01 | 0.88 | 0.47 | 320789.17 |
|  |  |  |  |  |  |  |  |  |
| **Log-normal** |  |  |  |  |  |  |  |  |
| Chemotherapy | 17188.04 |  | 0.65 |  |  | 0.45 |  |  |
| Pembrolizumab + Chemotherapy | 186704.02 | 169515.97 | 1.75 | 1.10 | 154305.90 | 1.04 | 0.59 | 286855.65 |

Abbreviation: Incr Cost=Incremental cost; Lys=life-years; Incr Lys=Incremental life-years; QALYs= Quality-adjusted life-years; Incr QALYs=Incremental Quality-adjusted life-years; ICER= Incremental cost-effectiveness ratio;
